# Influence of the regulatory peptide galanin on cytokine expression in human monocytes

**DOI:** 10.1111/nyas.14111

**Published:** 2019-05-10

**Authors:** Andrea Ramspacher, Magdalena Neudert, Andreas Koller, Sandra Schlager, Barbara Kofler, Susanne Maria Brunner

**Affiliations:** ^1^ Research Program for Receptor Biochemistry and Tumor Metabolism, Department of Pediatrics University Hospital of the Paracelsus Medical University Salzburg Austria; ^2^ University Clinic of Ophthalmology and Optometry, Research Program for Ophthalmology and Glaucoma Research University Hospital of the Paracelsus Medical University Salzburg Austria; ^3^ Department of Blood Group Serology and Transfusion Medicine University Hospital of the Paracelsus Medical University Salzburg Austria

**Keywords:** galanin, galanin receptor, monocyte, cytokine, chemokine

## Abstract

Current research into neuropeptides is bringing to light many remarkable functions of these endocrine/neurocrine/paracrine factors, such as their roles in modulating immune responses. Galanin is a neuropeptide expressed in both neural and non‐neural tissues and exerts its effects through three G protein–coupled receptors, GAL_1,2,3_‐R. It has been demonstrated that galanin has modulatory effects on immune cells, including neutrophils and natural killer cells. Because monocytes express GAL_2_‐R, and therefore are expected to be a target of galanin, we analyzed the effect of galanin on the expression of cytokines and chemokines by monocytes. Galanin increased the expression of IL‐1β up to 1.5‐fold, TNF‐α, IL‐10, IL‐18, and CCL3 up to twofold, and CXCL8 up to fourfold in nonactivated monocytes, but had no major effect on activated monocytes. A cross‐correlation analysis of cytokine expression profiles, irrespective of the activation status of the monocytes, revealed that galanin changed the cross‐correlation of the expression of certain cytokines. Galanin abolished several significant correlations in IFN‐γ–stimulated monocytes. For example, treatment with 10 nM galanin changed the Spearman's rank coefficient of IL‐18 and CXCL8 from 0.622 (*P* ≤ 0.01) to 0.126. These results further emphasize the importance of neuroregulatory peptides, such as galanin and their therapeutic potential to treat inflammatory diseases.

## Graphical abstract

Neuropeptides have various functions, including modulating immune responses. Because monocytes express one of the receptors for the neuropeptide galanin, the authors analyzed the effect of galanin on the expression of cytokines and chemokines by monocytes. Their data indicate that galanin modulates the immune responses of monocytes by regulating cytokine expression networks and that it exerts different functions in naive and inflammation‐induced cells.

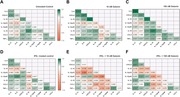

## Introduction

In recent decades, the discovery that there is bidirectional regulation between the neuroendocrine and the immune systems has brought regulatory neuropeptides into focus. This crosstalk between the two systems provides a finely orchestrated physiological and immunological reaction to pathogens.^1^, ^2^ The reciprocal effect is based on common receptors which respond to mediators of both the immune and neuroendocrine systems.^3^ However, pertubation of the sensitive balance between pro‐ and anti‐inflammatory neuropeptides and cytokines can result in autoimmune disease.^4^

The regulatory neuropeptide galanin and its three G protein–coupled transmembrane receptors (GAL_1_‐R, GAL_2_‐R, and GAL_3_‐R) are primarily expressed in the central and peripheral nervous systems.^5^ They regulate several physiological functions, such as nociception, feeding, and satiety.^6^ In addition, expression of galanin system components by various cell types of the immune system has already been well documented.^7^, ^8^, ^9^ In several animal models of inflammation, expression of GAL_1_‐R is elevated in peripheral tissue.^10^ In a porcine model of antral ulceration, elevation of GAL_1,2,3_‐R was observed in stomach tissue, accompanied by acute inflammation.^11^ In a transgenic mouse model of experimental autoimmune encephalomyelitis, overexpression of galanin led to abolishment of the disease, whereas a loss‐of‐function mutation in the galanin gene increased disease severity.^12^ Two *in vivo* studies conducted in our laboratory investigated the influence of GAL_3_‐R on different inflammatory diseases of the skin. Absence of GAL_3_‐R in an *in vivo* model of psoriasis considerably mitigated the course of the disease.^2^ Contrary to this finding, autoimmune arthritis induced in the same GAL_3_‐R knockout mouse line displayed increased severity.^13^ Therefore, these contrasting results indicate that the function of galanin in inflammatory processes is dependent on the type of inflammation. We recently reported on the function of the galanin system in diverse types of immune cells. For example, we detected GAL_2_‐R expression in both human and murine polymorphonuclear neutrophils (PMNs), and galanin treatment sensitized the response of PMNs toward CXCL8.^8^ Similarly, galanin treatment of natural killer (NK) cells enhanced their responsiveness to IL‐12 and IL‐18 and stimulated the production of IFN‐γ.^9^ These studies show a direct modulatory role of galanin on several different types of immune cells.

Monocytes are another important cell type in the early immune response. They are among the first cells to arrive at the site of infection. Upon antigen stimulation, they can either mature into macrophages or immediately release numerous cytokines to evoke immune responses. The latter molecules include potent proinflammatory cytokines, such as IL‐6, IL‐1β, IL‐18, and TNF‐α, and chemokines like CXCL8 and CCL3, which induce chemotaxis of cells of the innate and adaptive immune systems.^14^ Released IL‐12 induces the production of IFN‐γ by NK and T cells.^15^ IL‐12 is a heterodimeric cytokine composed of two subunits, IL‐12p35 and IL‐12p40. Together, they form the biologically active IL‐12p70. Counterbalancing these actions, monocytes can release high levels of the anti‐inflammatory cytokine IL‐10. Monocytes and macrophages also express neuroendocrine receptors and ligands^16^ and thus are susceptible to mediators of the neuroendocrine system.^1^ For example, neuropeptide Y interferes with the recruitment of monocytes to sites of inflammation.^17^, ^18^, ^19^ Vasoactive intestinal peptide effectively blocks neutrophil chemotaxis and infiltration by inhibiting NF‐κB–dependent CXCL8 gene expression.^20^

Although mRNA expression of galanin and GAL_2_‐R in human monocytes has been reported by us,^9^ there have not yet been any studies on monocytes and their responsiveness to galanin. Therefore, the aim of the present study was to investigate whether galanin has modulatory effects on primary human monocytes.

## Materials and methods

### Immune cell isolation and treatment

All immune cells were isolated from surplus buffy coat material from healthy donors (*n* = 32) who gave informed consent for the use of the surplus material for research purposes in accordance with the Helsinki Declaration and following the guidelines of the Salzburg State Ethics Research Committee. Buffy coats were generated and preselected based on the sedimentation rate, with a cutoff of 5% sedimentation after 1 h, and the ratio of neutrophils, lymphocytes, monocytes, eosinophils, and basophils was determined with a Sysmex XS‐800i (Sysmex, Kobe, Japan) system.^9^ Peripheral blood mononuclear cells were isolated by Ficoll‐Paque^TM^ (1.077 g/L) (GE Healthcare, Chicago, IL) gradient centrifugation. CD14^+^ monocytes were isolated by using the Human Pan Monocytes Isolation Kit (Miltenyi Biotec). Isolated cells were resuspended in RPMI‐1640 medium containing 10% fetal bovine serum (Thermo Fisher Scientific, Waltham, MA), 2 mM GlutaMAX^TM^ (Thermo Fisher Scientific, Austria), 10 mM HEPES (Sigma‐Aldrich, St. Louis, MO), and 1 × penicillin‐streptomycin‐amphotericin B mixture (Lonza, Basel, Switzerland) and seeded in a 24‐well plate (1 × 10^6^ cells/mL). After 1 h of rest, cells were treated with different concentrations of galanin (1 nM, 3 nM, 10 nM, 100 nM, and 1 µM) or with 100 ng/mL IFN‐γ, or with the combination of both, and incubated for 20 h (37 °C, 5% CO_2_). Depending on the number of isolated monocytes from each donor, galanin treatments did not always include all concentrations, which resulted in different *n* values in every independent experiment. Subsequently, cells were harvested in TRI Reagent® (Molecular Research Center, Inc., Cincinnati, OH) and subjected to RNA isolation.

### Determination of cytokine expression by quantitative PCR

RNA was isolated with TRI Reagent according to the manufacturer's instructions. Two micrograms of human RNA were used to generate cDNA by using MAXIMA^TM^ reverse transcriptase (Thermo Fisher Scientific, Austria) following the manufacturer's protocol. Expression levels of proinflammatory cytokines (IL‐12p35, IL‐12p40, TNF‐α, IL‐1β, IL‐18, and IL‐6), chemokines (CXCL8 and CCL3), and the anti‐inflammatory cytokine IL‐10 were quantified via qPCR using SYBR® Green SuperMix (BioRad, Hercules, CA). The amplification was performed for 40 cycles (97 °C for 15 s, 63 °C for 30 s, and 72 °C for 10 s) with specific primers for the genes of interest (Table S1, online only). Expression levels of all genes relative to the housekeeping gene *RPL27* were calculated by the ΔΔ quantification cycle (Cq) method using the formula:

(1) 2−(( Cq  of  the  gene  of  interest )−( Cq  of  the  housekeeping  gene  RPL 27)).

### Statistical analysis

The differences in mRNA expression between the IL‐12p70 positive and negative groups were analyzed by the Mann–Whitney U‐test. The differences in mRNA expression between every single galanin treatment and the corresponding control treatment were analyzed using the Wilcoxon matched‐pairs signed‐rank test. Relationships between the relative mRNA expression levels of the different cytokines were examined using the Spearman's rank correlation coefficient. Spearman's rank correlation coefficients ≤ –0.5 and ≥0.5 were considered as a strong correlation. *P* values <0.05 were considered statistically significant.

## Results

**Figure 1:**
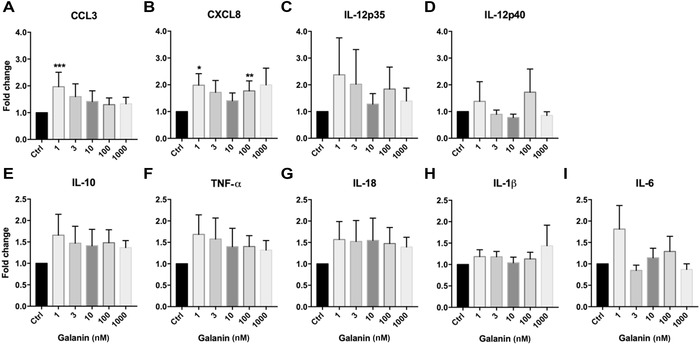
Relative change of expression of (A) CCL3, (B) CXCL8, (C) IL‐12p35, (D) IL‐12p40, (E) IL‐10, (F) TNF‐α, (G) IL‐18, (H) IL‐1β, and (I) IL‐6 upon treatment of human monocytes with galanin, compared with vehicle‐treated cells. The numbers of independent experiments were: 1 nM (*n* = 14), 3 nM (*n* = 17), 10 nM (*n* = 18), 100 nM (*n* = 18), and 1000 nM (*n* = 18). Values are presented as mean ± SEM. *P < 0.05, **P < 0.01 indicate significant fold‐difference of mRNA expression compared with vehicle‐treated cells.

To elucidate the effect of galanin on human monocytes, we isolated cells from the buffy coats of healthy human donors, subsequently treated the cells with different concentrations of galanin for 20 h, and then measured mRNA expression of the proinflammatory cytokines TNF‐α, IL‐12p35, IL‐12p40, IL‐1β, IL‐18, and IL‐6, the anti‐inflammatory cytokine IL‐10, and the proinflammatory chemokines CXCL8 and CCL3. We observed significant increases in the expression levels of CCL3 and CXCL8 upon galanin treatment (1 and 100 nM) compared with the vehicle‐treated controls (Fig. 1A and B). None of the other genes showed changes in mRNA expression upon galanin treatment.

A detailed analysis of vehicle‐treated control monocytes revealed high variability of cytokine expression among donors (Fig. S1, online only). Approximately half of the donors expressed no detectable levels of IL‐12p35, IL‐12p40, IL‐1β, IL‐6, and CXCL8, whereas the other half expressed high levels of the same cytokines (Fig. S1, online only). The most substantial variations were found for the subunits of the IL‐12 heterodimer, IL‐12p35 and IL‐12p40. To form active IL‐12 (IL‐12p70), both subunits are required.^15^ Therefore, we compared mRNA expression levels of IL‐12p35 and IL‐12p40 and classified donors according to their expression levels. Monocytes with detectable levels of IL‐12p35 and IL‐12p40 were classified as IL‐12p70–positive monocytes, and cells with no detectable levels of one subunit or both subunits, and hence lacking active IL‐12, were classified as IL‐12p70–negative monocytes (Table S1, online only).

**Figure 2:**
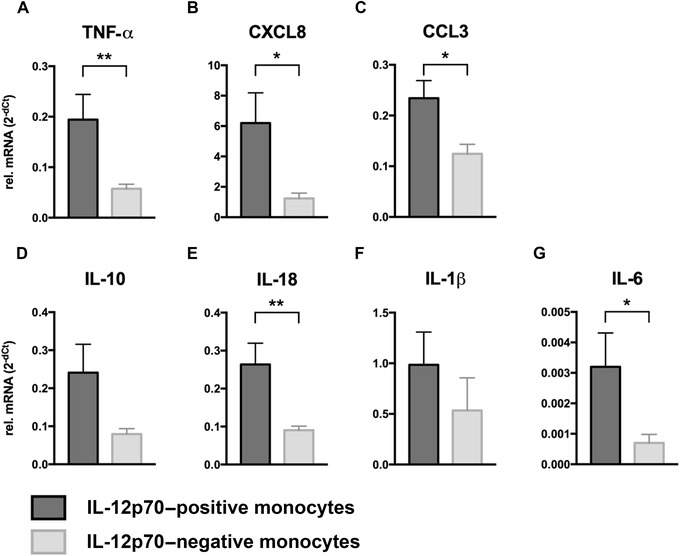
Cytokine mRNA levels of IL‐12p70–positive monocytes (*n* = 19) and IL‐12p70–negative monocytes (*n* = 13). Expression levels of both groups were compared for (A) TNF‐α, (B) CXCL8, (C) CCL3, (D) IL‐10, (E) IL‐18, (F) IL‐1β, and (G) IL‐6. Values are presented as mean ± SEM. *P < 0.05, **P < 0.01 indicate significant fold‐difference of mRNA expression compared with vehicle‐treated cells.

We observed consistently higher expression levels of all cytokines and chemokines in the IL‐12p70–positive monocyte group compared to the IL‐12p70–negative group (Fig. 2). Differences in the expression of TNF‐α, CXCL8, CCL3, IL‐18, and IL‐6 were statistically significant. The expression levels of IL‐10 and IL‐1β did not show significant differences, due to the fairly high interindividual variation of the isolated monocytes, but they tended to be lower in the IL‐12p70–negative group. As the IL‐12p70–positive and IL‐12p70–negative groups show differing expression levels of cytokines and chemokines under baseline conditions, we wondered whether these two groups also behave differently toward galanin treatment.

**Figure 3:**
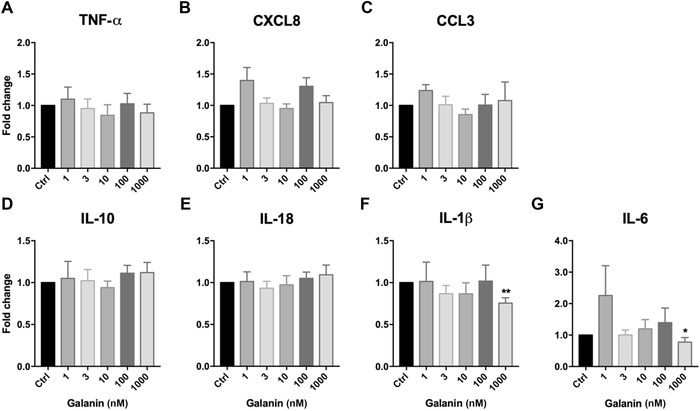
Relative change in the expression of (A) TNF‐α, (B) CXCL8, (C) CCL3, (D) IL‐10, (E) IL‐18, (F) IL‐1β, and (G) IL‐6 in human IL‐12p70–positive monocytes upon treatment with galanin, compared with vehicle‐treated cells. The numbers of independent experiments were: 1 nM (*n* = 4), 3 nM (*n* = 5), 10 nM (*n* = 9), 100 nM (*n* = 9), and 1000 nM (*n* = 10). Values are presented as mean ± SEM. *P < 0.05, **P < 0.01 indicate significant fold‐difference of mRNA expression compared with vehicle‐treated cells.

Accordingly, we stratified cytokine/chemokine mRNA expression of galanin‐treated monocytes into IL‐12p70–positive and IL‐12p70–negative categories and found that the response to galanin treatment differed between the two groups. In the IL‐12p70–positive monocytes, only the treatment with 1 µM galanin led to a reduction of mRNA expression of the proinflammatory cytokines IL‐1β and IL‐6 (Fig. 3F and G). The expression of all other cytokines and chemokines was not influenced by galanin in the IL‐12p70–positive monocytes.

**Figure 4:**
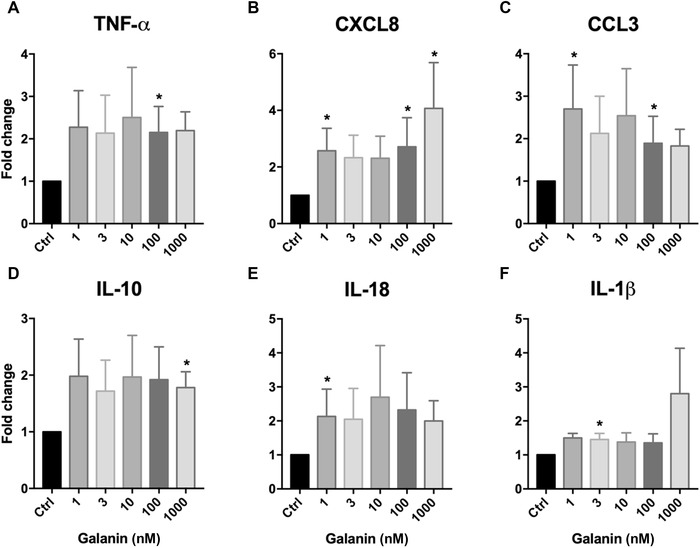
Relative change in the expression of (A) TNF‐α, (B) CXCL8, (C) CCL3, (D) IL‐10, (E) IL‐18, and (F) IL‐1β in human IL‐12p70–negative monocytes upon treatment with galanin, compared with vehicle‐treated cells. The numbers of independent experiments were: 1 nM (*n* = 10), 3 nM (*n* = 12), 10 nM (*n* = 9), 100 nM (*n* = 9), and 1000 nM (*n* = 8). Values are presented as mean ± SEM. *P < 0.05 indicates significant fold‐difference of mRNA expression compared with vehicle‐treated cells.

In contrast, treatment of the IL‐12p70–negative monocytes with galanin consistently led to increased mRNA expression of TNF‐α, CXCL8, CCL3, IL‐10, IL‐18, and IL‐1β (Fig. 4). Not all treatment groups reached statistical significance, again due to the high interindividual variability of cytokine and chemokine expression. However, treatment with 1 nM galanin led to a twofold increase in CXCL8 mRNA expression, a 2.5‐fold increase in CCL3 expression, and a twofold increase in IL‐18 expression. Treatment with 3 nM galanin significantly increased the mRNA expression of IL‐1β. Upon treatment with 100 nM galanin, we observed a twofold increase in TNF‐α mRNA expression and a twofold increase in CXCL8 and CCL3 expression. Treatment with 1 µM galanin led to a fourfold increase of mRNA expression of CXCL8 and a twofold increase of IL‐10 expression.

To elucidate if the modulating effect of galanin on cytokine expression is altered under inflammatory conditions, cells were treated with IFN‐γ (100 ng/mL) in combination with galanin for 20 h and then the cytokine expression profiles were compared with those of cells treated with IFN‐γ only. Overall, galanin treatment did not obviously affect the levels of cytokine expression of IFN‐γ–stimulated monocytes (Tables S3 and S4, online only).

In the present study, we only observed regulatory effects of galanin on naive monocytes when the cells were stratified by their IL‐12p70 expression. However, the scientific literature reports a complex network of regulatory mechanisms and correlations of cytokines and chemokines that orchestrate the immune response of a cell.^21^ Therefore, we wondered if galanin might act not on the expression of single cytokines but on the cytokine network in general. Consequently, we analyzed whether cytokine expression levels correlate with each other and if correlation patterns change upon galanin treatment. For this purpose, we calculated the Spearman's rank correlation coefficient for each cytokine and treatment, and subsequently compared the correlation coefficient of every pair of cytokines among treatments (galanin only, IFN‐γ only, and galanin + IFN‐γ). For this study, we decided to use galanin concentrations of 10 and 100 nM, which should resemble *in vivo* levels.

**Figure 5:**
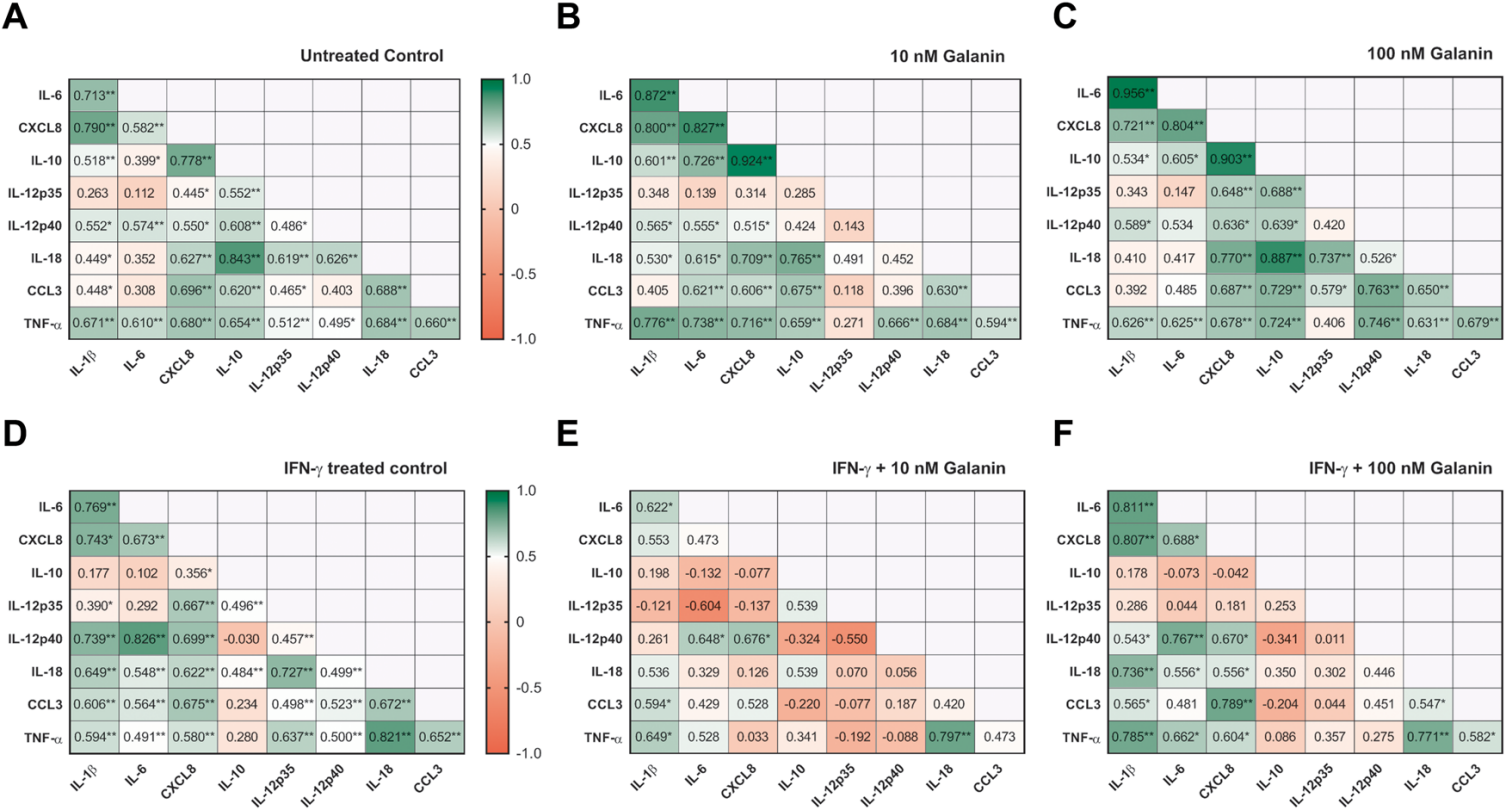
Spearman's rank correlation coefficients of relative cytokine mRNA expression levels of human monocytes: (A) untreated control, (B) treated with 10 nM galanin, (C) treated with 100 nM galanin, (D) treated with IFN‐γ, (E) treated with IFN‐γ + 10 nM galanin, and (F) treated with IFN‐γ + 100 nM galanin. *n* = 18. *P < 0.05, **P < 0.01 indicate significant fold‐difference of mRNA expression compared with vehicle‐treated cells.

The most significant correlation in vehicle‐treated monocytes was found between IL‐18 and IL‐10 (*R* = 0.843, *P* ≤ 0.01). The anti‐inflammatory cytokine IL‐10 also correlated with TNF‐α (*R* = 0.654, *P* ≤ 0.01), CCL3 (*R* = 0.620, *P* ≤ 0.01), IL‐12p35 (*R* = 0.552, *P* ≤ 0.01), and IL‐12p40 (*R* = 0.608, *P* ≤ 0.01). CXCL8 correlated with IL‐10 (*R* = 0.778, *P* ≤ 0.01), IL‐1β (*R* = 0.790, *P* ≤ 0.01), TNF‐α (*R* = 0.680, *P* ≤ 0.01), CCL3 (*R* = 0.696, *P ≤ *0.01), IL‐18 (*R* = 0.627, *P* ≤ 0.01), and the IL‐12 subunits IL‐12p35 (*R* = 0.445, *P* ≤ 0.05) and IL‐12p40 (*R* = 0.550, *P* ≤ 0.05). Other correlations included TNF‐α with IL‐1β (*R* = 0.671, *P* ≤ 0.01), IL‐6 (*R* = 0.610, *P* ≤ 0.01), CCL3 (*R* = 0.660, *P* ≤ 0.01), IL‐18 (*R* = 0.684, *P* ≤ 0.01), and both IL‐12p35 (*R* = 0.512, *P* ≤ 0.01) and IL‐12p40 (*R* = 0.495, *P* ≤ 0.05). IL‐6 correlated with IL‐1β (*R* = 0.713, *P* ≤ 0.01), IL‐12p40 (*R* = 0.574, *P* ≤ 0.01), and CXCL8 (*R* = 0.582, *P* ≤ 0.01). Other correlations were found between IL‐10 and IL‐1β (*R* = 0.518, *P* ≤ 0.01). IL‐18 correlated with IL‐12p35 (*R* = 0.619, *P* ≤ 0.01), with CCL3 (*R* = 0.688, *P* ≤ 0.01), and with IL‐12p40 (*R* = 0.626, *P* ≤ 0.01) (Fig. 5A).

Treatment with 10 nM galanin changed the correlation pattern significantly (Fig. 5B). Most of the significant correlations of IL‐12p35 were lost, including the ones with TNF‐α, CCL3, IL‐18, IL‐12p40, CXCL8, and IL‐10. In addition, the significant correlations between IL‐12p40 and IL‐18 and between IL‐12p40 and IL‐10 were lost, whereas the correlation coefficient increased between IL‐12p40 and TNF‐α (*R* = 0.666, *P* ≤ 0.01). Significant correlations were also induced between IL‐6 and CCL3 (*R* = 0.621, *P* ≤ 0.01) and IL‐18 (*R* = 0.615, *P* ≤ 0.05) upon treatment with 10 nM galanin.

Treatment with 100 nM galanin resulted in less changes in the correlation pattern (Fig. 5C). Significant correlations were lost between IL‐12p35 and TNF‐α and between IL‐12p35 and IL‐12p40, and another correlation that was lost was between IL‐12p40 and IL‐6. New significant correlations occurred between IL‐12p35 and CXCL8 (*R* = 0.648, *P* ≤ 0.01), and between IL‐12p40 and CXCL8 (*R* = 0.636, *P* ≤ 0.01), TNF‐α (*R* = 0.746, *P* ≤ 0.01), and CCL3 (*R* = 0.763, *P* ≤ 0.01).

When inflammatory conditions were simulated by treatment of monocytes with IFN‐γ, cytokine profiles shifted toward an increased proinflammatory state compared to untreated control cells (Fig. 5D). Thus, strong significant correlations were detected, particularly between proinflammatory cytokines. Strong correlations formed between IL‐1β and IL‐6 (*R* = 0.769, *P* ≤ 0.01), CXCL8 (*R* = 0.743, *P* ≤ 0.05), IL‐12p40 (*R* = 0.739, *P* ≤ 0.01), IL‐18 (*R* = 0.649, *P* ≤ 0.01), CCL3 (*R* = 0.606, *P* ≤ 0.01), and TNF‐α (*R* = 0.594, *P* ≤ 0.01). Other correlations were found between CXCL8 and IL‐6 (*R* = 0.673, *P* ≤ 0.01), IL‐12p35 (*R* = 0.667, *P* ≤ 0.01), IL‐12p40 (*R* = 0.699, *P* ≤ 0.01), IL‐18 (*R* = 0.622, *P* ≤ 0.01), CCL3 (*R* = 0.675, *P* ≤ 0.01), and TNF‐α (*R* = 0.580, *P* ≤ 0.01). The strongest correlation was found between IL‐6 and IL‐12p40 (*R* = 0.826, *P* ≤ 0.01), and IL‐6 also correlated with IL‐18 (*R* = 0.548, *P* ≤ 0.01) and CCL3 (*R* = 0.564, *P* ≤ 0.01). A strong correlation was also found between IL‐12p35 and IL‐18 (*R* = 0.727, *P* ≤ 0.01) and TNF‐α (*R* = 0.637, *P* ≤ 0.01). IL‐12p40 correlated with CCL3 (*R* = 0.523, *P* ≤ 0.01) and TNF‐α (*R* = 0.500, *P* ≤ 0.01). Furthermore, IL‐18 correlated with CCL3 (*R* = 0.672, *P* ≤ 0.01) and TNF‐α (*R* = 0.821, *P* ≤ 0.01), and CCL3 correlated with TNF‐α (*R* = 0.652, *P* ≤ 0.01).

Interestingly, combinatorial treatment of monocytes with IFN‐γ and 10 nM galanin changed the correlation profile dramatically (Fig. 5E). Many of the significant positive correlations of the IFN‐γ–only stimulated cells were lost or turned negative in the combined treatment (Fig. 5E). Two strong negative correlations were found between IL‐12p35 and IL‐6 (*R* = –0.604) and IL‐12p40 (*R* = –0.550). Other correlations were lost upon galanin treatment, for example, between IL‐12p35 and CXCL8 (*R* = –0.137), TNF‐α (*R* = –0.192), CCL3 (*R* = –0.077), and IL‐18 (*R* = 0.070). Strong, significant correlations were also lost between IL‐12p40 and IL‐1β (*R* = 0.261), TNF‐α (*R* = –0.088), CCL3 (*R* = 0.187), and IL‐18 (*R* = 0.056). Correlations between IL‐18 and IL‐6 (*R* = 0.329), CXCL8 (*R* = 0.126), and CCL3 (*R* = 0.420), between TNF‐α and CXCL8 (*R* = 0.033), and between CCL3 and IL‐10 (*R* = –0.220), IL‐6 (*R* = 0.429) and TNF‐α (*R* = 0.473) were also lost.

In contrast to the effects of IFN‐γ + 10 nM galanin, IFN‐γ + 100 nM galanin showed only slight differences compared to IFN‐γ alone (Fig. 5F). Reduced correlation coefficients were found between IL‐10 and CCL3 (*R* = –0.204) and TNF‐α (*R* = 0.086), and between IL‐12p35 and CXCL8 (*R* = 0.181), IL‐18 (*R* = 0.302), CCL3 (*R* = 0.044), and TNF‐α (*R* = 0.275). Correlations between IL‐12p40 and IL‐12p35 (*R* = 0.011) and TNF‐α (*R* = 0.275) were weakened as well, compared to IFN‐γ–only treatment.

## Discussion

Derived from common myeloid progenitor cells, monocytes, together with other cell types involved in innate immunity, form the first‐line immune response to pathogens. We previously demonstrated the modulatory effect that galanin has on various cells of the innate system, including neutrophils and NK cells. Here, after monocyte isolation and treatment with galanin, we found significant changes in the expression profiles of the chemokines CXCL8 and CCL3. Moreover, comparing the results of vehicle‐treated control monocytes from every donor, we found substantial variation in the levels of cytokine/chemokine mRNA expression.

The varying expression levels of cytokines and chemokines led us to postulate that the isolated monocytes might differ in their activation status. In particular, variable mRNA expression was found for the IL‐12 subunits, IL‐12p35 and IL‐12p40. These two subunits form biologically active IL‐12, which is one of the first cytokines expressed in phagocytic cells after pathogen contact. IL‐12 predominantly affects NK and T cells, induces the production of IFN‐γ and other proinflammatory cytokines, enhances cytotoxicity, and induces proliferation.^15^, ^22^ As IL‐12 is expressed in the early immune response, the expression levels of p35 and p40 give an indication of the activation status of monocytes. Hence, based on IL‐12p70 status, we classified isolated monocytes into IL‐12p70–positive monocytes and IL‐12p70–negative monocytes, resembling activated or nonactivated monocytes, respectively. In agreement with the activation status, IL‐12p70–positive monocytes showed higher expression levels of all cytokines investigated, compared with IL‐12p70–negative monocytes. The expression profile of IL‐12p70–positive monocytes is consistent with the expression profiles of monocytes activated with bacterial lipopolysaccharide.^23^

In previous studies, treatment with galanin did not directly affect immune cells; however, it modulated their sensitivity to cytokines and chemokines, and altered cytokine expression patterns.^8^, ^9^ Galanin treatment of IL‐12p70–positive (activated) monocytes did not affect cytokine mRNA expression, whereas the treatment of IL‐12p70–negative (nonactivated) monocytes did increase the mRNA expression of cytokines and shifted the cytokine expression profile toward an inflammatory state. However, these changes did not follow a clear dose–response relationship. Notably, the change of mRNA expression of CCL3 and IL‐18 tended to follow a bell‐shaped dose response. Bell‐shaped dose responses have, for example, been shown for the effect of galanin on edema formation in the skin.^24^ Despite the classification into IL‐12p70–positive and IL‐12p70–negative monocytes, the monocyte population within the two groups remains heterogeneous and still shows a high variability in response to galanin treatment, which can explain the diffuse dose–response curves. Contrary to our findings that galanin treatment shifted the cytokine expression profile toward an inflammatory state, decreased TNF‐α production upon galanin treatment was reported in microglia.^25^ A similar inhibitory effect of galanin on TNF‐α was demonstrated in *Staphylococcus aureus*–stimulated mouse macrophages.^26^ In the present study, the expression levels in nonactivated monocytes of the proinflammatory cytokines TNF‐α, IL‐1β, and IL‐18 and the chemokines CXCL8 and CCL3 were all significantly upregulated, even when galanin was present at low concentrations (1–3 nM). Very interestingly, alongside the proinflammatory cytokines, the very potent anti‐inflammatory cytokine IL‐10 was also upregulated by galanin. This bidirectional modulation of cytokine expression suggests that galanin plays an important role in the regulation of cytokine homeostasis. Immunomodulatory functions of galanin were also reported in NK cells and neutrophils.^8^, ^9^ Interestingly, stimulation of monocytes with IFN‐γ and galanin simultaneously did not alter the cytokine expression levels significantly compared to the treatment with IFN‐γ alone.

It is well established that cytokines act in a tight‐knit network. It is therefore essential to study the broad cytokine network rather than changes in the expression of single cytokines. Therefore, we hypothesized that galanin might influence the entire cytokine network and the interdependent regulation of its cytokine members.

As expected, the expression levels of several cytokines correlated with each other in naive monocytes. We further observed that physiological levels of galanin influence the complex interaction network of cytokines. The interdependency of the mRNA expression of these cytokines was previously reported in the literature. For example, IL‐18 regulates early mRNA production of TNF‐α and IL‐1β, followed by CXCL8.^27^ As galanin influences the IL‐18 interaction network, it could have an effect on this activation cascade. The anti‐inflammatory cytokine IL‐10 also exhibits stronger correlations with the proinflammatory cytokines CXCL8, TNF‐α, IL‐18, and IL‐1β upon galanin (10 nM) treatment of monocytes. IL‐10 expression in monocytes initiates inhibition of NF‐κB as a regulatory mechanism to decelerate inflammation.^28^ Furthermore, induction of IL‐10 happens only after several hours, whereas expression of proinflammatory cytokines is an immediate response. This also contributes to the balancing nature of IL‐10 and its inflammation‐buffering characteristic.^29^ We found reduced correlations of IL‐12p35 and IL‐12p40 with several other cytokines. As previously reported, IL‐10 is a potent inhibitor of IL‐12 expression, which could account for this reduction.^30^, ^31^ These findings indicate a modulatory function of galanin, maintaining and restoring cytokine homeostasis by bidirectional regulation to avoid a detrimental shift toward autoimmunity. However, a limitation of the present study is the lack of further time points due to limited availability of donor material.

When inflammatory conditions were induced by treatment of monocytes with IFN‐γ, the correlation pattern changed in favor of proinflammatory cytokines compared with the untreated control. Correlations of IL‐6, IL‐1β, and IL‐18 were stronger, in agreement with typical cytokine profiles of the early immune response,^23^, ^32^ whereas correlations of IL‐10 were partially lost. IL‐12p35 was not influenced by IFN‐γ stimulation substantially. Upon combinatorial stimulation with IFN‐γ and 10 nM galanin, the correlations involving IL‐10 weakened further. The same effect was observed for the correlations of IL‐12p35. This indicates that, under inflammatory conditions, galanin disrupts the balance between pro‐ and anti‐inflammatory cytokines. This could be a critical action to dampen inflammatory conditions by overexpression of anti‐inflammatory factors. Correlations of CCL3 with the proinflammatory cytokines IL‐6 and IL‐12 and the anti‐inflammatory cytokine IL‐10 decreased upon galanin treatment. One study reported a milder course of arthritis when a CCR1 antagonist was applied (CCR1 is a CCL3 receptor), and linked the expression of CCL3 to other chronic inflammatory diseases.^33^ Taking these findings together, galanin treatment weakens correlations of CCL3 with other cytokines, possibly to downregulate the acute immune response. *In vitro* studies reported an important role of CXCL8 in autoimmune diseases by its ability to induce transendothelial migration of neutrophils.^34^ We also observed decreases in the correlation coefficients between CXCL8 and IL‐18, CCL3, and IL‐10. This again indicates a possible regulatory function of galanin to avoid autoimmune reactions.

Our data indicate that galanin modulates the immune responses of monocytes by regulating cytokine expression networks. Furthermore, galanin exerts different functions in naive and inflammation‐induced cells. Changes in correlation patterns by galanin treatment do not elucidate the detailed role of galanin on monocytes, but they clearly illustrate the modulatory effect of galanin. Nonetheless, future studies need to address the functional consequences of galanin receptor activation in monocytes, both *in vitro* and *in vivo*. Our results further emphasize the importance of this regulatory neuropeptide in the immune response and establish its modulating effects on monocytes.

## Author contributions

M.N. and A.K. designed and performed experiments and analyzed data. A.R. analyzed data and wrote the manuscript. S.S. provided human patient samples. B.K. and S.M.B. obtained resources for the study, designed experiments, critically discussed the data, and coedited the manuscript. All authors approved the final version of the manuscript. All authors accept responsibility for the integrity of the data analyzed.

## Competing interests

The authors declare no competing interests.

## Supporting information


**Figure S1**. Cytokine mRNA levels of all isolated monocytes from individual donors (*n* = 18). Expression levels are shown for (A) TNF‐α, (B) IL‐12p35, (C) IL‐12p40, (D) IL‐1β, (E) IL‐6, (F) IL‐10, (G) IL‐18, (H) CCL3, and (I) CXCL8. Values are presented as mean ± SEM.

**Table S1**. Forward and reverse primers used in qRT‐PCR.

**Table S2**. mRNA expression levels of IL‐12 subunits p35 and p40.

**Table S3**. Relative change in expression levels of IL‐1β, IL‐6, IL‐10, IL‐12p35, IL‐12p40, IL‐18, CCL3, CXCL8, and TNF‐α in the IL‐12p70–positive monocyte group treated with IFN‐γ and 1 µM, 100 nM, 10 nM, 3 nM, or 1 nM galanin, compared with control treatment. The control treatment (no galanin) was set as 100%.

**Table S4**. Relative change in expression levels of IL‐1β, IL‐6, IL‐10, IL‐12p35, IL‐12p40, IL‐18, CCL3, CXCL8, and TNF‐α in the IL‐12p70–negative monocyte group treated with IFN‐γ and 1 µM, 100 nM, 10 nM, 3 nM, or 1 nM galanin, compared with control treatment. The control treatment (no galanin) was set as 100%.
