# Supplementary figures and images for: Influence of the regulatory peptide galanin on cytokine expression in human monocytes
Source: Ann N Y Acad Sci. 2019 May 10;1455(1):185–95. doi: 10.1111/nyas.14111 (PMC6899851; doi:10.1111/nyas.14111)

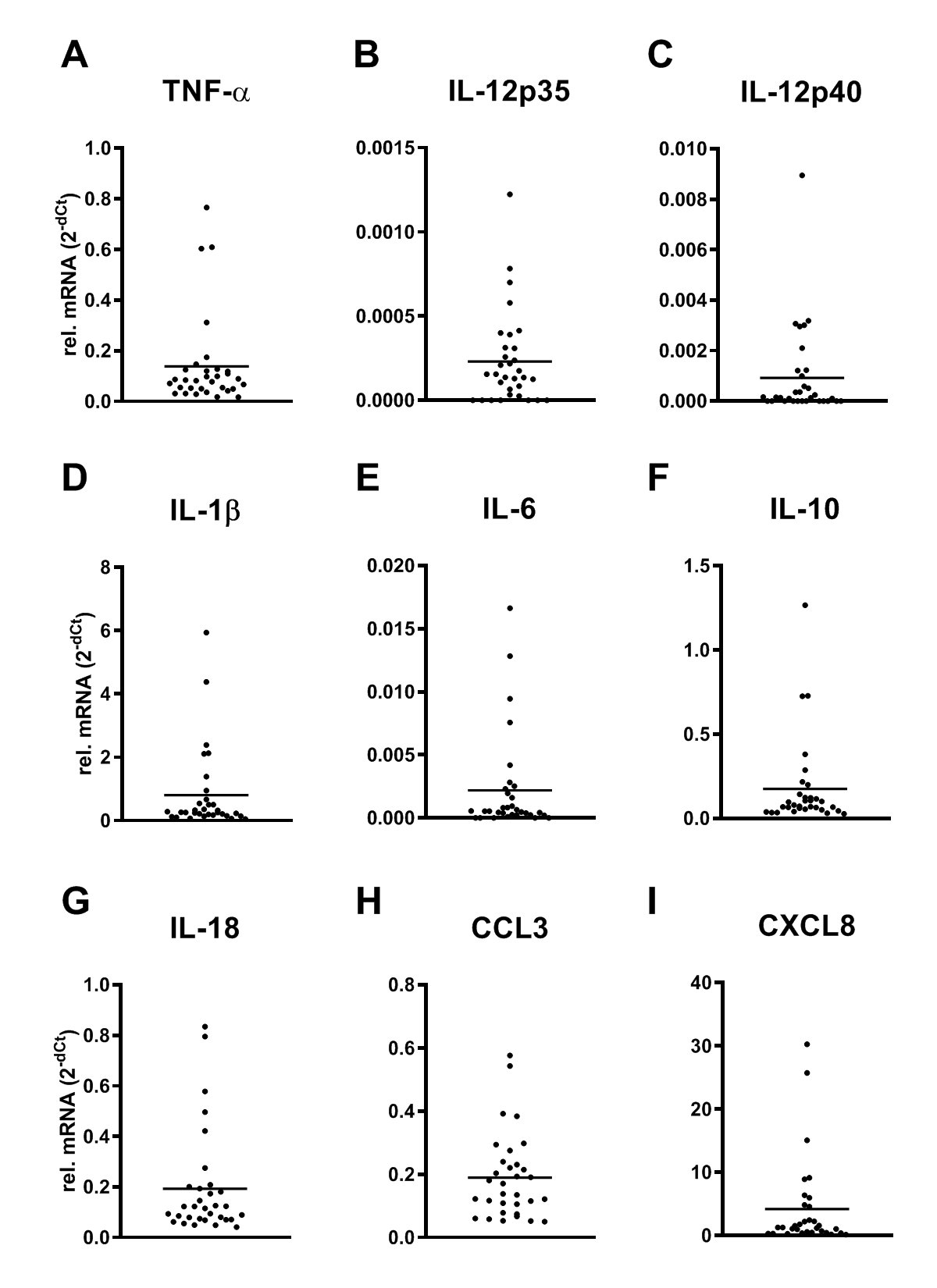

Supplement: Supplementary file 1 — Figure S1. Cytokine mRNA levels of all isolated monocytes from individual donors (n = 18). Expression levels are shown for (A) TNF‐α, (B) IL‐12p35, (C) IL‐12p40, (D) IL‐1β, (E) IL‐6, (F) IL‐10, (G) IL‐18, (H) CCL3, and (I) CXCL8. Values are presented as mean ± SEM. [file NYAS-1455-185-s001.tif]
