# Supplementary material for: Influence of the regulatory peptide galanin on cytokine expression in human monocytes
Source: Ann N Y Acad Sci. 2019 May 10;1455(1):185–95. doi: 10.1111/nyas.14111 (PMC6899851; doi:10.1111/nyas.14111)
Supplement: Supplementary file 2 — Table S1. Forward and reverse primers used in qRT‐PCR. [file NYAS-1455-185-s002.docx]

| Gene | *Forward (5’* -> *3’)* | *Reverse (5’* -> *3’)* |
| --- | --- | --- |
| *CCL3* | CATGGCTCTCTGCAACCAGTTCT | CCGGCTTCGCTTGGTTAGGAAG |
| *CXCL8* | TGACTTCCAAGCTGGCCGTG | TTCTGTGTTGGCGCAGTGTGG |
| *IL-1β* | AGGCTGCTCTGGGATTCTCTTCA | CCATCCAGAGGGCAGAGGTCCA |
| *IL-6* | AGATGTAGCCGCCCCACACAG | CCAGTGCCTCTTTGCTGCTTTCA |
| *IL-10* | GACCCAGACATCAAGGCGCA | CATTCTTCACCTGCTCCACGGC |
| *IL-12p35* | GTTCCCATGCCTTCACCACTCC | GGCCTCCACTGTGCTGGTTTTA |
| *IL-12p40* | TGGACTCTCCGTCCTGCCCA | ACCACCATTTCTCCAGGGGCAT |
| *IL-18* | CCAGATCGCTTCCTCTCGCA | TCCAGGTTTTCATCATCTTCAGCTA |
| *TNF-α* | CCTGCTGCACTTTGGAGTGA | CTTGTCACTCGGGGTTCGAG |
| *RPL27* | GCTGGAATTGACCGCTACC | TCTCTGAAGACATCCTTATTGACG |

**Table S1. Forward and reverse primers used in qRT-PCR**
