# Supplementary material for: Influence of the regulatory peptide galanin on cytokine expression in human monocytes
Source: Ann N Y Acad Sci. 2019 May 10;1455(1):185–95. doi: 10.1111/nyas.14111 (PMC6899851; doi:10.1111/nyas.14111)
Supplement: Supplementary file 3 — Table S2. mRNA expression levels of IL‐12 subunits p35 and p40. [file NYAS-1455-185-s003.docx]

| **Donor** | **IL-12 p35** (rel. mRNA) | **IL-12p40** (rel. mRNA) |  |
| --- | --- | --- | --- |
| **23** | 0.00031 | 0.00013 | **IL-12p70 positive monocytes** |
| **22** | 0.00024 | 0.00013 |  |
| **20** | 0.00041 | 0.00025 |  |
| **4** | 0.00022 | 0.00016 |  |
| **29** | 0.00014 | 0.0001 |  |
| **8** | 0.00078 | 0.0012 |  |
| **2** | 0.00058 | 0.0012 |  |
| **12** | 0.00016 | 0.00035 |  |
| **17** | 0.0000661 | 0.00015 |  |
| **21** | 0.0000334 | 0.0001 |  |
| **25** | 0.00011 | 0.00036 |  |
| **6** | 0.00013 | 0.00052 |  |
| **5** | 0.0007 | 0.003 |  |
| **1** | 0.0004 | 0.0021 |  |
| **3** | 0.0012 | 0.0089 |  |
| **10** | 0.00013 | 0.001 |  |
| **15** | 0.00039 | 0.0032 |  |
| **32** | 0.00016 | 0.003 |  |
| **14** | 0.00014 | 0.003 |  |
| **13** | n.d. | 0.00059 | **IL-12p70 negative monocytes** |
| **7** | 0.00002531 | n.d. |  |
| **9** | 0.00026 | n.d. |  |
| **18** | 0.0000845 | n.d. |  |
| **19** | 0.00018 | n.d. |  |
| **24** | 0.00031 | n.d. |  |
| **26** | 0.00021 | n.d. |  |
| **11** | n.d. | n.d. |  |
| **16** | n.d. | n.d. |  |
| **27** | n.d. | n.d. |  |
| **28** | n.d. | n.d. |  |
| **30** | n.d. | n.d. |  |
| **31** | n.d. | n.d. |  |

**Table S2. mRNA expression levels of IL-12 subunits p35 and p40**

n.d. = not detectable.
