# Supplementary material for: Influence of the regulatory peptide galanin on cytokine expression in human monocytes
Source: Ann N Y Acad Sci. 2019 May 10;1455(1):185–95. doi: 10.1111/nyas.14111 (PMC6899851; doi:10.1111/nyas.14111)
Supplement: Supplementary file 4 — Table S3. Relative change in expression levels of IL‐1β, IL‐6, IL‐10, IL‐12p35, IL‐12p40, IL‐18, CCL3, CXCL8, and TNF‐α in the IL‐12p70–positive monocyte group treated with IFN‐γ and 1 µM, 100 nM, 10 nM, 3 nM, or 1 nM galanin, compared with control treatment. The control treatment (no galanin) was set as 100%. [file NYAS-1455-185-s004.docx]

| Gene | IFN-γ | IFN-γ +  1 µM Gal | IFN-γ +  100 nM Gal | IFN-γ +  10 nM Gal | IFN-γ +  3 nM Gal | IFN-γ +  1 nM Gal |
| --- | --- | --- | --- | --- | --- | --- |
| **IL-1β** | 100% | 97.8%  ± 12.4% | 75.6%  ± 13.3%* | 71.1%  ± 14.8% | 79.1%  ± 11.1% | 58.1%  ± 13.7% |
| **IL-6** | 100% | 105.8%  ± 11.4% | 75.5%  ± 11.4% | 89.8%  ± 19.8% | 85.8%  ± 12.8% | 64.7%  ± 19.4% |
| **IL-10** | 100% | 121.6%  ± 17.7% | 87.7%  ± 11.4% | 89.6%  ± 13.9% | 86.8%  ± 14.3% | 164.7%  ± 66.1% |
| **IL-12p35** | 100% | 100.8%  ± 13.1% | 109.8%  ± 14.7% | 107.2%  ± 21.8% | 98.5%  ±10.1% | 688.1%  ± 611.9% |
| **IL-12p40** | 100% | 115.2%  ± 18.7% | 93.2%  ± 22.4% | 78.9%  ± 17.6% | 104.7%  ± 23.3% | 58.8%  ± 25.2% |
| **IL-18** | 100% | 99.3%  ± 7.9% | 76.3%  ± 10.1% | 75.1%  ± 18.3% | 90.9%  ± 10.1% | 65.7%  ± 17.5% |
| **CCL3** | 100% | 98.5%  ± 11.9% | 85.5%  ± 7.9% | 93.3%  ± 10.4% | 85.8%  ± 9.9% | 92.2%  ± 5.6% |
| **CXCL8** | 100% | 132.7%  ± 18.9% | 112.5%  ± 26.6% | 91.3%  ± 15.7% | 92.1%  ± 11.5% | 91.3%  12.8% |
| **TNF-α** | 100% | 94.8%  ± 20.1% | 82.6%  ± 14.4% | 68.2%  ± 14.1% | 90.1%  ± 14.7% | 56.9%  ± 12.4% |

**Table S3. Relative change in expression levels of IL-1β, IL-6, IL-10, IL-12p35, IL-12p40, IL-18, CCL3, CXCL8 and TNF-α in the IL-12p70 positive monocyte group treated with IFN-γ and 1 µM, 100 nM, 10 nM, 3 nM or 1 nM galanin compared to control treatment; the control treatment (no galanin) was set as 100 %.**

The values are presented as mean ± SEM. * p < 0.05.
