# Supplementary material for: Influence of the regulatory peptide galanin on cytokine expression in human monocytes
Source: Ann N Y Acad Sci. 2019 May 10;1455(1):185–95. doi: 10.1111/nyas.14111 (PMC6899851; doi:10.1111/nyas.14111)
Supplement: Supplementary file 5 — Table S4. Relative change in expression levels of IL‐1β, IL‐6, IL‐10, IL‐12p35, IL‐12p40, IL‐18, CCL3, CXCL8, and TNF‐α in the IL‐12p70–negative monocyte group treated with IFN‐γ and 1 µM, 100 nM, 10 nM, 3 nM, or 1 nM galanin, compared with control treatment. The control treatment (no galanin) was set as 100%. [file NYAS-1455-185-s005.docx]

| Gene | IFN-γ | IFN-γ +  1 µM Gal | IFN-γ +  100 nM Gal | IFN-γ +  10 nM Gal | IFN-γ +  3 nM Gal | | IFN-γ +  1 nM Gal | |  |
| --- | --- | --- | --- | --- | --- | --- | --- | --- | --- |
| **IL-1β** | 100% | 123%  ± 23.4% | 121.3%  ± 38.1% | 102.3%  ± 17.9% | | 96.3%  ± 21.6% | | 109.3%  ± 21.8% | |
| **IL-6** | 100% | 97.5%  ± 12.5% | 107.8%  ± 20.1% | 95.9%  ± 11.8% | | 60.7%  ± 8.6%  * | | 97.1%  ± 5.2% | |
| **IL-10** | 100% | 106.2%  ± 14.4% | 122.5%  ± 15.4% | 89.5%  ± 5.0% | | 97.6%  ± 13.4% | | 104.8%  ± 13.7% | |
| **IL-12p35** | 100% | 106.0%  ± 9.5% | 83.9%  ± 26.0% | 129.4%  ± 10.8% | | 117.9%  ± 21.3% | | 111.0%  ± 30.0% | |
| **IL-12p40** | 100% | 147.4%  ± 41.3% | 127.1%  ± 54.4% | 113.2%  ± 23.8% | | 96.2%  ± 24.1% | | 95.0%  ± 25.8% | |
| **IL-18** | 100% | 112.8%  ± 8.5% | 101.5%  ± 17.1% | 98.4%  ± 5.5% | | 90.9%  ± 11.4% | | 108.7%  ± 5.9% | |
| **CCL3** | 100% | 89.7%  ± 7.5% | 90.9%  ± 11.6% | 135.6%  ± 12.4% | | 101.8%  ± 8.5% | | 122.3%  ± 14.5% | |
| **CXCL8** | 100% | 103.1%  ± 20.2% | 138.8%  ± 50.4% | 124.3%  ± 24.9% | | 101.2%  ± 18.6% | | 137.2%  ± 28.7% | |
| **TNF-α** | 100% | 113.4%  ± 6.9% | 112.5%  ± 13.6% | 90.8%  ± 12.4% | | 85.8%  ± 13.3% | | 96.7%  ± 13.4% | |

**Table S4. Relative change in expression levels of IL-1β, IL-6, IL-10, IL-12p35, IL-12p40, IL-18, CCL3, CXCL8 and TNF-α in the IL-12p70 negative monocyte group treated with IFNγ and 1 µM, 100 nM, 10 nM, 3 nM or 1 nM galanin compared to control treatment; the control treatment (no galanin) was set as 100%.**

The values are presented as mean ± SEM. * p < 0.05.
